# C-MAC Video Laryngoscope versus Conventional Direct Laryngoscopy for Endotracheal Intubation During Cardiopulmonary Resuscitation

**DOI:** 10.3390/medicina55060225

**Published:** 2019-05-29

**Authors:** Byeong Chul Min, Jong Eun Park, Gun Tak Lee, Tae Rim Kim, Hee Yoon, Won Chul Cha, Tae Gun Shin, Keun Jeong Song, Minsu Park, Heewon Han, Sung Yeon Hwang

**Affiliations:** 1 Department of Emergency Medicine, Samsung Medical Center, Sungkyunkwan University School of Medicine, Seoul 06351, Korea; byeongchul.min@samsung.com (B.C.M.); qtjongeun@naver.com (J.E.P.); guntak.lee@samsung.com (G.T.L.); taerimi.kim@samsung.com (T.R.K.); wildhi.yoon@samsung.com (H.Y.); docchaster@gmail.com (W.C.C.); tackles@naver.com (T.G.S.); kj64.song@samsung.com (K.J.S.); 2 Statistics and Data Center, Samsung Medical Center, Seoul 06351, Korea; minsu51.park@samsung.com (M.P.); heewon.han@sbri.co.kr (H.H.)

**Keywords:** airway management, tracheal intubation, direct laryngoscopy, video laryngoscope, cardiac arrest

## Abstract

*Background and objectives*: To compare the first pass success (FPS) rate of the C-MAC video laryngoscope (C-MAC) and conventional Macintosh-type direct laryngoscopy (DL) during cardiopulmonary resuscitation (CPR) in the emergency department (ED). *Materials and Methods*: This study was a single-center, retrospective study conducted from April 2014 to July 2018. Patients were categorized into either the C-MAC or DL group, according to the device used on the first endotracheal intubation (ETI) attempt. The primary outcome was the FPS rate. A multiple logistic regression model was developed to identify factors related to the FPS. *Results*: A total of 573 ETIs were performed. Of the eligible cases, 263 and 310 patients were assigned to the C-MAC and DL group, respectively. The overall FPS rate was 75% (*n* = 431/573). The FPS rate was higher in the C-MAC group than in the DL group, but there was no statistically significant difference (total *n* = 431, 79% compared to 72%, *p* = 0.075). In the multiple logistic regression analysis, the C-MAC use had higher FPS rate (adjusted odds ratio: 1.80; 95% CI, 1.17–2.77; *p* = 0.007) than that of the DL use. *Conclusions*: The C-MAC use on the first ETI attempt during cardiopulmonary resuscitation in the emergency department had a higher FPS rate than that of the DL use.

## 1. Introduction

Although airway management is a key component of cardiopulmonary resuscitation (CPR), the optimal strategy for airway management during CPR remains unknown [1,2]. Nevertheless, endotracheal intubation (ETI) is frequently performed during CPR in the emergency department (ED). ETI during CPR potentially protects the lungs against aspiration of vomitus or secretions, ensures adequate ventilation and oxygenation, minimizes gastric insufflation, and allows ventilation without pausing chest compressions [3].

Recently, video laryngoscope (VL) has been widely used to facilitate ETI in the ED [4]. Studies comparing the usefulness of VL with direct laryngoscopy (DL) in various situations in the ED have been published. Although it has produced conflicting results, existing literature supports VL use in the ED [5,6,7]. However, the usefulness of VL during CPR in the ED has not been widely evaluated. In addition, all but one of the previous studies focused on the GlideScope^®^ video laryngoscope (GVL); the one focused on the heterogeneous types of VL [8,9,10,11].

ETI during CPR deserves special attention and needs more research because it is particularly challenging due to suboptimal intubating conditions, such as glottis movement in accordance with chest compressions and a limited pre-assessment of the airway. Performance of each VL may vary depending on specific clinical circumstances and patient airway conditions [12,13]. In this perspective, the device can affect ETI success rate during CPR. Therefore, a comparison of ETI success between the specific VL, which is widely used in the ED, and conventional DL would be clinically relevant. In this study, the first pass success (FPS) rate of the C-MAC video laryngoscope (C-MAC) and conventional Macintosh-type DL during CPR in the ED were compared.

## 2. Materials and Methods

This retrospective study was conducted in the ED of Samsung Medical Center (a tertiary university affiliated referral hospital with over 70,000 visits to the ED annually) from April 2014 to July 2018. This institution has an accredited four-year emergency medicine residency program. Approximately 350–400 ETIs are performed per year among adult patients in the ED. The study was approved by the institutional review board of Samsung Medical Center (IRB number, 2018-12-106); informed consent was waived because the study was retrospective and required no intervention. All methods were performed in accordance with relevant guidelines/regulations.

Patients who fulfilled all of the following criteria were eligible for analysis: (1) Eighteen years or older who underwent CPR in the ED, (2) ETI due to cardiac arrest (CA), and (3) C-MAC (Karl Storz Endoskope, Tuttlingen, Germany) or conventional DL use on the first ETI attempt. Cases involving ETI devices other than C-MAC and conventional DL on the first ETI attempt were excluded.

All ETI cases in the ED are registered in our institution’s registry [14]. All ETI processes were monitored by an independent medical staff who did not participate in the procedure. The registry is completed by the operator and the medical staff after the ETI procedure. The following data were obtained from the airway registry and electronic medical records: Patient demographics, which included age, sex, body mass index, and past medical history; CA site, initial rhythm, time to ETI after CPR initiation, survival data; indication for ETI, number of ETI attempts, devices to facilitate ETI, Cormack-Lehane (C-L) grade, presence of difficult airway characteristics; ETI-related complications, which included esophageal intubation (EI) and dental injury; and operator training level.

The patients were categorized into either the C-MAC group or the DL group, according to the device used on the first ETI attempt. A Macintosh-type blade (size three or four) or D-BLADE (Karl Storz Endoskope, Tuttlingen, Germany) was used in the C-MAC group; whereas, a conventional Macintosh blade (size three or four) was used in the DL group.

An ETI attempt was defined as the placement of a laryngoscope blade into the mouth, regardless of an endotracheal tube insertion attempt [14]. The FPS was defined as a successful ETI on the first attempt. Difficult airway characteristics for laryngoscopy were determined based on only external features, such as short neck, facial trauma, obesity, and cervical immobilization, because full assessment of the airway was not feasible during CPR. Glottic visualization was described using a C-L classification reported by the operator.

The primary outcome was the FPS rate. The secondary outcomes were glottic visualization, multiple attempts rate, and ETI-related complications, which included EI and dental injury, return of spontaneous circulation, 24-hour mortality, and survival-to-discharge.

The continuous variables were described using medians with interquartile ranges (IQR), and the Wilcoxon rank sum test was used for comparisons. Categorical data were described using numbers with percentages, and the chi-square test or Fisher’s exact test was appropriately used for comparisons. A multiple logistic regression model was developed to assess variables that were related to the FPS. Potential confounding variables were selected as a priority by clinical plausibility. The following variables were included in the final model: Device used for ETI (C-MAC compared to DL) on the first attempt, patient age, sex, body mass index, indication for ETI (non-traumatic compared to traumatic), CA site, operator training level, and presence of difficult airway characteristics. The results were presented as adjusted odds ratios (aOR) with a 95% confidence interval (CI). The Hosmer-Lemeshow test was used to assess goodness-of-fit of the model. *P*-values less than 0.05 were considered statistically significant for all statistical tests. STATA 15.0 (STATA Corporation, College Station, TX, USA) and SAS 9.4 (SAS Institute Inc., Cary, NC, USA) were used for all statistical analyses.

## 3. Results

### 3.1. Baseline Characteristics

A total of 607 ETIs were performed for adult patients with CA during the study period. Of these, 34 were excluded from the study, and 573 ETIs were analyzed. Of the eligible cases, 263 and 310 patients were assigned to the C-MAC and DL group, respectively. The baseline characteristics of each group are presented in Table 1. The median age of the patients was 67 (53–78) years; 65% of the patients were males. Age, sex, BMI, and comorbidities of the patients were not significantly different between the groups. Out-of-hospital CA was more frequent in the C-MAC group than in the DL group (91% compared to 82%, *p* = 0.003).

### 3.2. ETI-Related Data

ETI-related data are shown in Table 2. The number of patients who experienced traumatic arrests was higher in the C-MAC group than in the DL group (total *n* = 44, 13% compared to 3%, *p* < 0.001). Difficult airway characteristics for laryngoscopy were more frequently observed in the C-MAC group than in the DL group (total *n* = 80, 17% compared to 11%, *p* = 0.025). The operator level on the first attempt was significantly different (*p* < 0.001).

### 3.3. Outcomes

**Figure 1:**
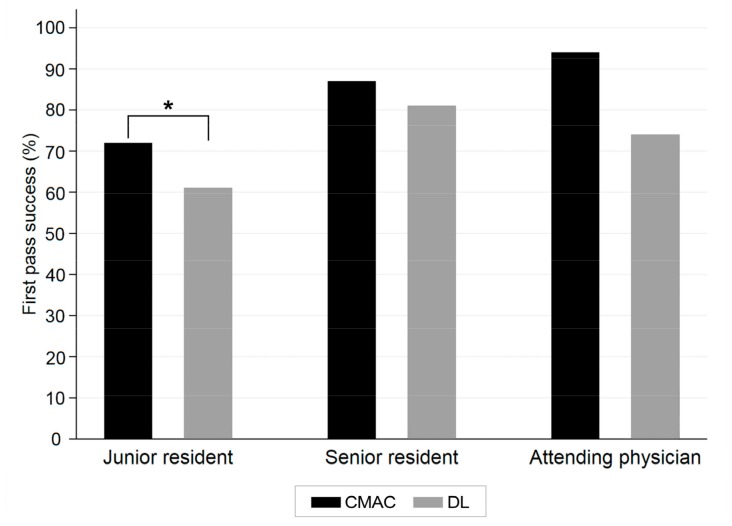
First pass success rate according to the status of the operator. C-MAC, C-MAC video laryngoscope; DL, conventional direct laryngoscopy. * *p*-value < 0.05.

The primary and secondary outcomes are shown in Table 3. The overall FPS rate was 75% (*n* = 431/573). The FPS rate was higher in the C-MAC group than in the DL group, but there was no statistically significant difference (total *n* = 431, 79% compared to 72%, *p* = 0.075). According to the operator status, the FPS rate was significantly higher in the C-MAC group than in the DL group, when the operator was a junior resident (total *n* = 268, C-MAC compared to DL, 111/154 (72%) compared to 69/114 (61%), *p* = 0.046), but not a senior resident (total *n* = 245, C-MAC compared to DL, 79/91 (87%) compared to 124/154 (81%), *p* = 0.207) or an attending physician (total *n* = 60, C-MAC compared to DL, 17/18 (94%) compared to 31/42 (74%), *p* = 0.067) (Figure 1).

Glottic visualization, which was indicated by C-L grade 1 or 2 on the first attempt, was better in the C-MAC group than in the DL group (total *n* = 449, 85% compared to 73%, *p* = 0.001). EI was more frequent in the DL group than in the C-MAC group (total *n* = 37, 9% compared to 4%, *p* = 0.017); however, there was no unrecognized EI in both groups. Return of spontaneous circulation and prognosis, including 24 h mortality and survival-to-discharge, were not significantly different between the groups.

### 3.4. Multivariable Analysis

In the multiple logistic regression analysis, FPS was higher in C-MAC use (aOR: 1.80; 95% CI, 1.17–2.77; *p* = 0.007) than in DL use (Table 4). Male patients (aOR: 0.49; 95% CI: 0.32–0.77; *p* = 0.002) and presence of difficult airway characteristics (aOR: 0.35; 95% CI: 0.16–0.74; *p* = 0.006) were associated with decreased FPS. Senior residents (aOR: 2.99; 95% CI: 1.91–4.71; *p* < 0.001) and attending physicians (aOR: 2.65; 95% CI: 1.52–6.81; *p* = 0.002) had increased FPS than that of junior residents.

## 4. Discussion

In this study, the efficacies of C-MAC and DL during CPR in the ED were evaluated. The C-MAC use on the first ETI attempt had a higher FPS than the DL use, after adjustment for confounding factors. In the C-MAC group, glottic visualization was significantly better than that of the DL group, whereas the EI was significantly less in the C-MAC group than in the DL group.

The most efficient device to facilitate ETI during CPR in the ED remains unclear. Only few studies have focused on this issue. Park et al. revealed that among novice emergency physicians, GVL use was associated with a better FPS rate and a shorter duration of chest compression pause than conventional DL was, during CPR in the ED [9]. In another study conducted in the same hospital, Kim et al. demonstrated that FPS, ETI speed, and ETI-related complications were not different between GVL and conventional DL among experienced operators during CPR in the ED [10]. Okamoto et al. used large data from a multicenter, prospective study of ED patients with CA to compare FPS between DL and VL, including C-MAC, McGrath, Airway Scope, and GVL [11]. Their study revealed that ETI with VL had a significantly higher FPS rate, better glottic visualization, and lower EI than DL use.

Given that ETI during CPR is difficult, international guidelines recommended that it should be performed only by trained personnel with a high level of skill and confidence [2]. However, a significant number of ETIs for patients with CA has been performed by less-experienced physicians in the real world [11]. In this study, approximately half of the ETIs were performed by junior residents. Overall, the FPS rate was higher in the C-MAC group than in the DL group, regardless of training level, but a statistically significant difference was only observed in the case of junior residents. These findings are consistent with those of previous studies [9,10,11]. This suggests that the benefits from C-MAC use are pronounced for less-experienced operators than for experienced operators. The experienced operators seem to be less affected by the type of device when performing ETI during CPR, because they are sufficiently familiar and have experience handling various devices. EM residents require training to establish competency in ETI to cope with a variety of situations. Our findings suggest that C-MAC may have more advantages for training less-experienced physicians for ETI during CPR, while the safety of the patient is less compromised.

There are possible explanations for the association between increased FPS rate and C-MAC use. Firstly, one of the benefits of the C-MAC is that, like other VLs, the supervisor and operator can share the same view on the monitor, so the supervisor can guide the operator and verify placement of the tubes into the trachea. This also seems to have caused the EI to be lower in the C-MAC group than in the DL group. Secondly, a previous study showed that C-MAC reduced the need for adjunctive maneuvers, including external laryngeal manipulation and gum-elastic bougie [15]. In addition, C-MAC usually uses a Macintosh–type blade with a conventional curvature that is familiar to emergency physicians. The curved blade compresses the tongue base and permits a direct approach to the glottis. Putting these together, these might make the ETI process easier for the operator.

Several factors might have affected the low FPS rate in this study. Firstly, any insertion of a laryngoscope blade into the mouth, whether or not the operator intended to insert a tube into the trachea, was strictly counted by a dedicated medical staff in real-time to eliminate recall and reporting bias [14]. Kerrey et al. reported that the first attempt failure during ETI was much more common than previously reported in a pediatric ED, when data collected by video review were analyzed [16]. Secondly, interruption of chest compressions for ETI attempts were strongly limited. This is because in most situations, chest compression should be prioritized over ETI during CPR. It is generally difficult to achieve successful ETI during chest compressions.

In this study, the FPS rate was affected by patient sex; males were significantly associated with a decrease in the FPS rate. One possible explanation for the result is that the proportion of obesity in males was higher than that in females. Obesity has been considered to be a risk factor for difficult laryngoscopy [17]. Another potential explanation might be the anatomical differences between males and females, such as soft tissues in the neck and fat distribution [18]. These findings should be taken into consideration when attempting ETI in patients with CA.

There are several limitations to be considered in this study. Firstly, it was a single-center study conducted in an urban, academic ED, so generalizability may be limited for other ED settings. Secondly, most of the data were collected during and immediately after CPR, but some were retrospectively obtained from medical records. Thirdly, the choice of device on the first attempt was at the discretion of the operator and availability of C-MAC blades. It was difficult to clearly identify the factors that affect the clinicians’ choice of device. Since operators’ preferences for devices were subjective and ETI process was entirely the responsibility of operators, the choice of device for ETI could not be controlled. Additionally, as our ED had only three C-MAC blades during the study period and blade sterilization took one day, there were times when the device was not available. This could have led to selection bias. Fourthly, data on chest compression pause time during the first ETI attempt were not presented. We have been strongly limiting the interruption of chest compressions for ETI attempts. Lastly, various types of blades, including Macintosh-type and hyperangulated D-BLADE are available for the C-MAC system currently; however, the shapes of the blades were not differentiated.

## 5. Conclusions

C-MAC use on the first ETI attempt during CPR in the ED had a higher FPS than that of DL use. In addition, when C-MAC was used as the first device, the glottic visualization was significantly improved, while the EI was significantly decreased as compared with the cases when DL was used. Our study might be useful to establish airway management strategies during CPR in the ED.

## Figures and Tables

**Table 1:** Comparison of baseline patient characteristics.

	Total (*n* = 573)	C-MAC (*n* = 263)	DL (*n* = 310)	*p*-Value
Patient Age (years)	67 (53, 78)	67 (52, 79)	67 (53, 78)	0.797
Patient Sex (male)	370 (65)	170 (65)	200 (65)	0.976
Patient BMI (kg/m^2^)				0.077
Normal (18.5–24.9)	62 (11)	21 (8)	41 (13)	
Underweight (<18.5)	368 (64)	169 (64)	199 (64)	
Obese (≥25)	143 (25)	73 (28)	70 (23)	
Patient Medical History				
Diabetes	130 (23)	59 (22)	71 (23)	0.894
Hypertension	187 (33)	75 (29)	112 (36)	0.053
Asthma	13 (2)	3 (1)	10 (3)	0.095
COPD	19 (3)	8 (3)	11 (4)	0.736
Congestive Heart Failure	22 (4)	6 (2)	16 (5)	0.074
Coronary Artery Disease	69 (12)	33 (13)	36 (12)	0.732
Chronic Kidney Disease	41 (7)	14 (5)	27 (9)	0.117
Stroke	38 (7)	14 (5)	24 (8)	0.246
Liver cirrhosis	12 (2)	5 (2)	7 (2)	0.766
Malignancy	120 (21)	50 (19)	70 (23)	0.295
Arrest Site				0.001
Out-of-hospital	495 (86)	241 (92)	254 (82)	
In-hospital	78 (14)	22 (8)	56 (18)	
Initial Rhythm, Shockable	65 (12)	24 (10)	41 (14)	0.269

Data are presented as medians with interquartile ranges or *n* (%). C-MAC, C-MAC video laryngoscope; DL, conventional direct laryngoscopy; BMI, body mass index; C-L grades, Cormack-Lehane grades; COPD, chronic obstructive pulmonary disease; CPR, cardiopulmonary resuscitation.

**Table 2:** Endotracheal intubation-related data.

	Total (*n* = 573)	C-MAC (*n* = 263)	DL (*n* = 310)	*p*-Value
Indication of Intubation				<0.001
Non-traumatic	529 (92)	229 (87)	300 (97)	
Traumatic	44 (8)	34 (13)	10 (3)	
Presence of Difficult Airway Characteristics	80 (14)	46 (17)	34 (11)	0.025
Operator Level on the First Attempt				<0.001
Junior Resident *	268 (47)	154 (59)	114 (37)	
Senior Resident ^†^	245 (43)	91 (35)	154 (50)	
Attending Physician	60 (10)	18 (7)	42 (14)	
Time to ETI, (min) ^‡^	2 (1, 4)	2 (1, 4)	2 (1, 4)	0.805

Data are presented as n (%). * Junior resident refers to first-year and second-year residents. ^†^ Senior resident refers to third-year and fourth-year residents. ^‡^ Time from chest compression to confirmation of tube placement into the trachea. C-MAC, C-MAC video laryngoscope; DL, conventional direct laryngoscopy; ETI, endotracheal intubation.

**Table 3:** Primary and secondary outcomes.

	Total (*n* = 573)	C-MAC (*n* = 263)	DL (*n* = 310)	*p*-Value
First Pass Success	431 (75)	207 (79)	224 (72)	0.075
C-L Grade 1 or 2 on the First Attempt	449 (78)	223 (85)	226 (73)	0.001
Multiple Attempts (≥3)	45 (8)	15 (6)	30 (10)	0.078
Esophageal Intubation	37 (6)	10 (4)	27 (9)	0.017
Dental Injury	5 (0.9)	1 (0.4)	4 (1)	0.381
ROSC	290 (51)	128 (49)	162 (52)	0.392
24-hour Mortality	436 (76)	203 (77)	233 (75)	0.571
Survival-to-Discharge	68 (12)	33 (13)	35 (12)	0.643

Data are presented as n (%). C-MAC, C-MAC video laryngoscope; DL, conventional direct laryngoscopy; C-L grade, Cormack-Lehane grade; ROSC, Return of spontaneous circulation.

**Table 4:** Univariable and multivariable analyses of factors associated with the first pass success.

	Univariable			Multivariable		
	OR	95% CI	*p*-Value	OR	95% CI	*p*-Value
Device on the First Attempt						
C-MAC (vs. DL)	1.42	0.96–2.09	0.075	1.80	1.17–2.77	0.007
Patient Age (65 years or more)	1.20	0.82–1.75	0.350	1.01	0.66–1.54	0.964
Patient Sex (male)	0.56	0.37–0.86	0.008	0.49	0.32–0.77	0.002
Patient BMI (kg/m^2^)				-	-	-
Normal (18.5–24.9)	Reference					
Underweight (<18.5)	1.21	0.64	0.559	1.21	0.61–2.37	0.588
Obese (≥25)	1.22	0.78–1.93	0.385	1.20	0.74–1.94	0.466
Nontraumatic Arrest (vs. Traumatic Arrest)	1.46	0.75–2.84	0.263	0.66	0.25–1.77	0.410
OHCA (vs. IHCA)	1.32	0.78–2.24	0.302	2.04	1.13–3.69	0.019
Operator Level on First Attempt						
Junior Resident *	Reference					
Senior Resident ^†^	2.36	1.55–3.59	0.000	2.99	1.91–4.71	<0.001
Attending Physician	1.96	0.99–3.87	0.054	2.65	1.52–6.81	0.002
Presence of Difficulty Airway Characteristics	0.52	0.32–0.86	0.011	0.35	0.16–0.74	0.006

* Junior resident refers to first-year and second-year residents. ^†^ Senior resident refers to third-year and fourth-year residents. *p*-value of Goodness-of-fit with the Hosmer–Lemeshow test = 0.140. OR, odds ratio; CI, confidence interval; C-MAC, C-MAC video laryngoscope; DL, conventional direct laryngoscopy; BMI, body-mass index; OHCA, out-of-hospital cardiac arrest; IHCA, in-hospital cardiac arrest.
